# Links between depressive symptoms and unmet health and social care needs among older prisoners

**DOI:** 10.1093/ageing/afv171

**Published:** 2016-01-13

**Authors:** Kate O'Hara, Katrina Forsyth, Roger Webb, Jane Senior, Adrian Jonathan Hayes, David Challis, Seena Fazel, Jenny Shaw

**Affiliations:** 1 School of Languages, Law and Social Sciences, Dublin Institute of Technology, Dublin, Ireland; 2 Offender Health Research Network, University of Manchester, Manchester, UK; 3 Institute of Brain Behaviour and Mental Health, University of Manchester, Manchester, UK; 4 University of Warwick—Medicine, Medical Teaching Centre, Coventry CV4 7AL, UK; 5 School of Nursing, Midwifery and Social Work, University of Manchester, Manchester, UK; 6 Department of Psychiatry, University of Oxford, Warneford Hospital, Oxford, UK

**Keywords:** ageing prisoners, depression, unmet needs, prison entry, older people

## Abstract

**Background:** absolute numbers of older prisoners and their proportion of the total prison population are increasing. They have multiple health and social care needs that are prominent on entry into prison. No previous studies have identified older prisoners' health and social care needs at this crucial point.

**Objective:** to examine unmet health and social care needs among older men entering prison and their links with depressive symptoms.

**Methods:** a cross-sectional survey across nine prisons in the North of England was completed. One hundred male prisoners aged between 60 and 81 were interviewed, using the Camberwell Assessment of Need—Forensic short version (CANFOR-S) and Geriatric Depression Scale—Short Form (GDS-15). Descriptive statistics were generated and *χ*^2^ tests performed.

**Results:** participants reported high levels of unmet needs as measured with the CANFOR-S, notably in the domains of knowledge about their condition and treatment (38%); psychological distress (34%); daytime activities (29%); benefits (28%); food (22%) and physical health (21%). The mean total number of unmet needs was 2.74, with a median of 2.0. More than half the sample (56%, 95% CI 45–66%) exhibited clinical signs of depression. A significant association between depressive symptomology and an unmet physical health need, as measured by the CANFOR-S, was detected (*χ*^2^ = 6.76, df = 1, *P* < 0.01).

**Conclusions:** high levels of depressive symptoms were experienced by older prisoners on entry into prison. Personalised health and social care needs assessment and discrete depression screening are required on prison entry to facilitate effective management of unmet needs.

## Introduction

An ageing population, changes to sentencing practices, and advances in obtaining forensic evidence have resulted in a striking increase in the number of older prisoners across the developed world [1–4]. In England and Wales, as of December 2014, there were 11,431 prisoners aged 50 and over, representing 13.5% of the overall prison population [5], a proportion that has steadily increased over the last two decades.

Older prisoners' health needs are greater than both their peers living in the community and those of younger prisoners [4, 6, 7]. Over 80% of older prisoners have at least one major illness [8] and, compared with younger prisoners, are significantly more likely to have received treatment in prison for a medical condition [4]. Hayes *et al.* [2] reported that over a third of older prisoners had some level of functional need in activities of daily living, with accommodation being the greatest unmet need (35%). Fazel *et al.* [9] reported that 77% of older prisoners were being prescribed medication, most frequently for cardiovascular problems. The most common mental health diagnosis among this group is depression (12–34%) [8, 10, 11]. Seventy per cent of older prisoners reported receiving treatment or counselling for a health problem in the year before prison entry [4]. Cooney *et al.* [12] reported that prison staff perceived older prisoners to have a physical health status 10 years older than people of the same biological age living in the community, acquiring age-related health problems 10 to 15 times faster than their peers in the general population [13–15]. Prison-related factors, including the stress of imprisonment, increased anxiety, isolation and separation from family may also be associated with lower life expectancy and premature ageing [16].

It is known that transitions between institutional healthcare settings and other care sectors are problematic for older people [17, 18], and there is evidence to suggest that this is similar for older people entering prison. Crawley and Sparks [19] identified a ‘state of trauma’ in early imprisonment, during which older prisoners struggled to cope with the stark changes to their physical environment. Mental illness symptom prevalence is highest during initial periods of custody [20–22].

According to Fazel *et al.* [8], older prisoners are at greater risk of depression if they report other types of ill-health. Murdoch, Morris and Holmes [23] found that, in prisoners over 55, chronic illness such as ischaemic heart disease and hypertension, among other conditions, resulted in higher depressive symptom scores, as measured by the GDS. To date, the only published research that has examined older prisoners' health and social care needs, or levels of depressive symptomology, specifically at point of entry into prison has been an overarching programme of work completed by the current authors of which these data form part. The current paper specifically investigates, using standardised assessments of health and social needs, whether there is an association between depressive symptomology and unmet health and social care needs. An association between depressive symptoms and unmet needs, even without evidence of causation, will be relevant to healthcare policy and practice for this vulnerable group across the UK and, potentially, more widely.

## Method

### Setting

The study was carried out in the northern regions of England. Nine local prisons were included, which incarcerated people awaiting trial, convicted of short sentences and those at the beginning of longer sentences. The sample included three private prisons and six public prisons. Prisons were located in both rural and urban areas. The National Research Ethics Service Committee West Midlands, Staffordshire reviewed and approved the project (09/H120347).

### Participants

All prisoners aged 60 or over who were newly received into the prisons from court were eligible for inclusion in the study. A consecutive sample of 100 participants was obtained between February 2010 and December 2011. We applied this minimum cut-off age as it was the most commonly used in the psychiatric literature when the study was designed [6, 9] and is in line with the cut-off age generally used by social services in the UK [24]. The age at which a prisoner is regarded as older is ‘inevitably arbitrary’ [3], with research studies use varying definitions including 50 [16], 55 [23] and 60 [6] years.

### Procedures

All participants completed structured assessments between 2 and 10 weeks after entry into prison; interviews lasted ∼30 min. The initial approach was made by the Older Prisoner Liaison Officer, or other designated staff member within each prison. Consent was obtained by one of two research assistants at least 24 h before assessments were administered. Questions contained in both assessments were read out loud to each participant. Further details of the study design and methods can be found elsewhere [24]. Assessments included the Geriatric Depression Scale short form (GDS-15) [25] which had a Cronbach alpha score of 0.85 suggesting good internal consistency. The GDS-15 contains 15 yes/no questions; items indicative of depression carry a score of 1. A total scale score of >5 is suggestive of depressive symptomology requiring further investigation [25]. The Camberwell Assessment of Need—Short Forensic Version [26] was also administered. The CANFOR-S measures health and social need experienced over the last month across 25 domains. Each domain is scored as follows: no need, met need, unmet need or not applicable. Ten per cent of interviews were scored by both research assistants to assess inter-rater reliability.

### Data analysis

Data were entered into the Statistical Package for the Social Sciences (SPSS) version 19 (SPSS Inc., 2011) by a research assistant and checked by a senior research assistant. Initial descriptive statistics were computed with further *χ*^2^ analysis undertaken to identify associations between unmet need and a GDS-15 score of >5 (indicating the presence of depressive symptomatology) [25]. All participants completed the CANFOR-S, while 14 participants declined to complete the GDS-15. The 95% confidence intervals (CIs) were calculated using the Wilson method, which prevents the lower confidence limit falling below zero where the percentage value is very small.

## Results

### Demographic and criminogenic characteristics

Participants' ages ranged between 60 and 81 years (*n* = 100; mean = 65.5; SD = 5.35 years). Nineteen per cent were incarcerated for violent crimes; 28% for crimes of a sexual nature; and 36% for other crimes including fraud (10%), acquisitive crimes (4%) and drug offences (7%). Seventeen per cent refused to disclose their offence. One quarter (25%) were serving sentences of <12 months and over half (51, 51%) had not been in prison previously. A large proportion of participants (43%) were housed on a vulnerable prisoner wing (VPW), a section of a prison that houses certain categories of prisoner considered to be at risk of harm from the general prison population.

### The CANFOR-S: unmet health and social care needs in older prisoners entering prison

The mean total of needs was 5.24 with a median of 5 (SD = 2.81). The mean total number of unmet needs was 2.74 with a median of 2.0 (SD = 2.65; range 0–15). The most common unmet needs were in the domains of information about health condition and treatment (38%); psychological distress (34%); daytime activities (29%); benefits (28%), food (22%) and physical health (21%). Only 1% had no needs (met or unmet) at all.

### The association between depression and unmet need in older prisoners

**Figure 1.**
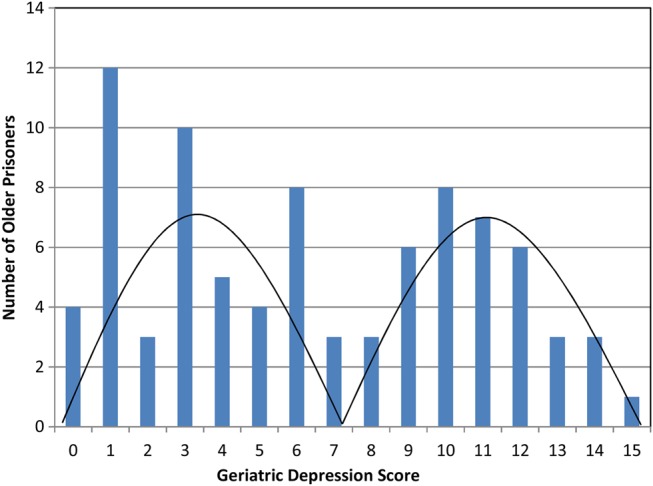
Distribution of GDS-15 scores (*n* = 86).

Geriatric depression scale scores were non-normally distributed and, as shown in Figure 1, a bimodal distribution was observed. Fifty-five per cent (95% CI 44–65%; *n* = 47) of the sample reached the cut-off of greater than five indicating clinically significant symptoms of depression; 23% (95% CI 14–32%; *n* = 20) scored >10, most likely indicating depressive symptoms of a higher level of severity.

Across all CANFOR-S domains, there was a consistent pattern of a statistically significant higher likelihood of unmet need among prisoners identified as having clinically significant depressive symptomatology, as seen in Table 1.

**Table 1.** Prevalence of clinically significant depressive symptomatology (GDS-15 score >5) according to rising number of unmet needs

Number of unmet needs	Total number of prisoners (*N* = 100)	GDS-15 score >5	%	(95% CI)
	*N*	*n*		
None	19	2	11	(3–24)
1–2	35	11	31	(16–46)
3–4	30	20	67	(49–83)
5 or more	16	14	88	(71–103)

A series of *χ*^2^ analyses investigated associations between GDS-15 scores >5, versus lower scores and the most prevalent unmet needs identified by the CANFOR-S (Table 2). All those with an unmet need regarding support for psychological distress exhibited depressive symptoms. There was a significant association between depression and an unmet need for daytime activity. Seventy-six per cent who had an unmet daytime activity need displayed clinically significant depressive symptomology. Of those with an unmet physical health need, 80% presented with depressive symptoms. This contrasts with those exhibiting no symptoms of depression, of whom only 20% had an unmet physical health need.

**Table 2.** Associations between clinically significant depressive symptomatology (GDS-15 score >5) and unmet health and social care need

GDS-15 stratum and unmet need association^b^	Unmet need	No unmet need	*χ*^2^ test
	*n* (%)	*n* (%)	
GDS-15 versus information about condition and treatment			*χ*^2^ = 4,38, df = 1, *P* < 0.05
No depressive symptoms [GDS-15 score 0–5]	9 (30)	30 (54)	
Any depressive symptoms [GDS-15 score 6–15]	21 (70)	26 (40)	
GDS-15 versus psychological distress			*χ*^2^ = 32.66, df = 1, *P* < 0.001
No depressive symptoms [GDS-15 score 0–5]	0 (0)	39 (66)	
Any depressive symptoms [GDS-15 score 6–15]	27 (100)	20 (34)	
GDS-15 versus daytime activities			*χ*^2^ = 5.2, df = 1, *P* < 0.05
No depressive symptoms [GDS-15 score 0–5]	5 (24)	34 (52)	
Any depressive symptoms [GDS-15 score 6–15]	16 (76)	31 (48)	
GDS-15 versus benefits			*χ*^2^ = 4.97, df = 1, *P* < 0.05
No depressive symptoms [GDS-15 score 0–5]	6 (25)	33 (53)	
Any depressive symptoms [GDS-15 score 6–15]	18 (75)	29 (47)	
GDS-15 versus food			*χ*^2^ = 6.10, df = 1, *P* < 0.05
No depressive symptoms [GDS-15 score 0–5]	5 (23)	34 (53)	
Any depressive symptoms [GDS-15 score 6–15]	17 (77)	30 (47)	
GDS-15 versus physical health			*χ*^2^ = 6.76, df = 1, *P* < 0.01
No depressive symptoms [GDS-15 score 0–5]	4 (20)	35 (53)	
Any depressive symptoms [GDS-15 score 6–15]	16 (80)	31 (47)	
GDS-15 versus money			*χ*^2^ = 4.63, df = 1, *P* < 0.05
No depressive symptoms [GDS-15 score 0–5]	2 (17)	37 (50)	
Any depressive symptoms [GDS-15 score 6–15]	10 (83)	37 (50)	
GDS-15 versus company			*P* = 0.03^a^
No depressive symptoms [GDS-15 score 0–5]	0 (0)	39 (49)	
Any depressive symptoms [GDS-15 score 6–15]	6 (100)	41 (51)	
GDS-15 versus treatment			*P* = 0.02^a^
No depressive symptoms [GDS-15 score 0–5]	0 (0)	39 (49)	
Any depressive symptoms [GDS-15 score 6–15]	7 (100)	40 (51)	
GDS-15 versus alcohol			*P* = 0.02^a^
No depressive symptoms [GDS-15 score 0–5]	0 (0)	39 (49)	
Any depressive symptoms [GDS-15 score 6–15]	7 (100)	40 (51)	
GDS-15 versus self-care			*P* = 0.06^a^
No depressive symptoms [GDS-15 score 0–5]	0 (0)	39 (48)	
Any depressive symptoms [GDS-15 score 6–15]	5 (100)	42 (52)	
GDS-15 versus transport			*P* = 0.06
No depressive symptoms [GDS-15 score 0–5]	0 (0)	39 (48)	
Any depressive symptoms [GDS-15 score 6–15]	5 (100)	42 (52)	

^a^Fisher's exact test.

^b^Other CANFOR-S needs tested: Telephone; Accommodation; Looking after the living environment; Intimate relationships; Basic education; Psychotic symptoms; Safety to self; Sexual expression; Sexual offending.

Eighty-three per cent of those with an unmet need around money and finances exhibited symptoms of depression. Similarly, there were significant associations between depressive symptomology and unmet treatment, company and alcohol needs; all participants with unmet needs in these three domains showed symptoms of depression.

## Discussion

### Main findings

High levels of depressive symptoms on entry into prison were found, with almost one in three older people reaching the cut-off score of >5 on the GDS-15, indicating symptoms of clinical depression.

All of those reporting an unmet need in the domain of psychological distress as measured by the CANFOR-S displayed clinically significant depressive symptomatology. This was also identified in the domains of company, treatment, alcohol, self-care and transport. A strong association between depressive symptomology and unmet physical health need was detected. It is unknown whether unmet physical health needs lead to depression, whether the opposite is true, or whether the presence of depression masks the reporting, recognition and subsequent health service response to physical ill-health. Therefore, a causal relationship cannot be determined. This requires further exploration.

### Comparison with existing evidence

Our study found that older people newly received into custody, either convicted, or on pre-trial detention had a higher mean number of unmet needs (∼3) than Hayes *et al.* [2] reported at later stages of incarceration (∼2). The most common type of unmet needs also differed between the two studies. Hayes and colleagues [2] reported that the most common unmet needs were accommodation, physical health and food, whereas in the current study they were information about a health condition and treatment, psychological distress and daytime activities. The lower rates of unmet need regarding psychological distress and information about a health condition or treatment, observed in those further into their period of imprisonment, optimistically points to prison-based healthcare services being able to address these concerns with some success, given time. Conversely, unmet physical health needs were lower in the current study of newly received prisoners compared with the Hayes *et al.* [2] study, raising the possibility that physical health may decline during incarceration for this group.

Over half of our sample (55%) had high rates of depressive symptomology, supporting findings from previous prison research, as well as studies in the community [27, 28]. Older prisoners who report being in poor health are at greater risk of being diagnosed with depression or presenting with depressive symptoms [8, 23], which is supported by the strong association between the presence of depressive symptomology and unmet physical health need that we observed. This is also the case among community samples in England [27].

Murdoch, Morris and Holmes [23] explored depression in elderly life-sentenced prisoners aged 55 and over using the standard GDS and reported that 48% scored within the mild depression range and 3% scored within the more severe depression range. In contrast, our study found lower rates of mild depressive symptoms, but higher rates of more severe depressive symptomology scores. This may suggest that older prisoners newly received into prison are more likely to experience more severe depressive symptoms than those at other points in the sentence, supporting previous research indicating that the initial entry period is particularly risky in terms of prisoners' mental health [29].

### Limitations

Only local prisons within reasonable geographical reach of the research base at the University of Manchester were selected for inclusion. Nonetheless, the characteristics of the study sample were not dissimilar to national statistics contemporaneous to the data collection period in relation to older prisoners' offences or ethnicity [3]. We used a continuous measure of depressive symptoms; the GDS-15 is not a diagnostic test, and hence, our results should be read with this in mind.

The study's small sample size of 100 is also a limitation, as well as missing GDS-15 data from 14 participants. Findings must be interpreted with caution, although results from this study may prompt further investigation and can be used to design larger confirmatory studies.

### Implications

The high rates of multiple unmet health and social care needs among older prisoners that we observed support the recommendation in the Department of Health's guidance [30] that older prisoners' health and social care needs should be systematically identified and addressed on entry. Currently, no standardised assessments exist that are specifically designed for older prisoners' needs. Senior *et al.* [24] confirmed that 81% of adult prison establishments in England and Wales had yet to establish specialised assessments for older people on prison entry.

The high prevalence of depression among older prisoners, now confirmed across a number of studies, supports the need for routine, effective and early depression screening to be established. The systematic use of health and social care assessments and subsequent care planning, alongside screening for depression, should provide a two-pronged approach to better addressing the high levels of depression among older prisoners with unmet health and social care needs.

This study has estimated prevalence of depressive symptomology and its possible association with poor physical health, and other unmet social needs in the vulnerable older prisoner population. The physical and mental health problems of older people are intertwined and the risk of depression is amplified by physical ill-health, thus further research is required to understand this complex relationship, to best inform policy and practice.

## Conclusion

Older prisoners experience an elevated number of multiple unmet needs on entry into prison. These are different to those experienced at other stages in their incarceration, indicating the need for an early, responsive, approach from a wide range of health, custodial and social support services throughout their imprisonment. Future research is required to fully understand this relationship and how services could best respond. Systematic health and social care needs assessments such as the Older prisoner Health and Social Care Assessment and Plan (OHSCAP), an assessment tool developed by the research group as part of the wider NIHR study [24], and/or the CANFOR-S, and depression screening using the GDS should be routinely administered. These tools are freely available and are quick to administer. Optimal treatment is required for older prisoners at prison entry and throughout their time in custody.

**Key points**

- There is a high prevalence of depressive symptomatology in older prisoners on prison entry.
- A strong relationship between older prisoners' unmet physical health needs and clinical symptoms of depression was detected.
- There is a need to systematically assess and manage older prisoners' health and social care needs and screen for depression on prison entry and throughout their incarceration.
- Further research in this area is required to fully inform practice.

## Conflicts of interest

None declared.

## Funding

The research reported in this paper was funded by the HS&DR programme or one of its proceeding programmes as project number 08/1809/230.^24^ S.F. is funded by the Wellcome Trust part of a Senior Research Fellowship in Clinical Science (095806).
